# Administration of granulocyte-colony stimulating factor accompanied with a balanced diet improves cardiac function alterations induced by high fat diet in mice

**DOI:** 10.1186/s12872-015-0154-6

**Published:** 2015-12-03

**Authors:** Pâmela Santana Daltro, Paula Santana Alves, Murilo Fagundes Castro, Carine M. Azevedo, Juliana Fraga Vasconcelos, Kyan James Allahdadi, Luiz Antônio Rodrigues de Freitas, Bruno Solano de Freitas Souza, Ricardo Ribeiro dos Santos, Milena Botelho Pereira Soares, Simone Garcia Macambira

**Affiliations:** Center for Biotechnology and Cell Therapy, Hospital Sao Rafael, Salvador, BA Brazil; Gonçalo Moniz Research Center, Oswaldo Cruz Foundation (CPqGM/FIOCRUZ), Salvador, BA Brazil; Federal University of Bahia, Salvador, BA Brazil

**Keywords:** Diabetes, Diabetic cardiomyopathy, Obesity, G-CSF

## Abstract

**Background/Objectives:**

High fat diet (HFD) is a major contributor to the development of obesity and cardiovascular diseases due to the induction of cardiac structural and hemodynamic abnormalities. We used a model of diabetic cardiomyopathy in C57Bl/6 mice fed with a HFD to investigate the effects of granulocyte-colony stimulating factor (G-CSF), a cytokine known for its beneficial effects in the heart, on cardiac anatomical and functional abnormalities associated with obesity and type 2 diabetes.

**Methods:**

Groups of C57Bl/6 mice were fed with standard diet (*n* = 8) or HFD (*n* = 16). After 36 weeks, HFD animals were divided into a group treated with G-CSF + standard diet (*n* = 8) and a vehicle control group + standard diet (*n* = 8). Cardiac structure and function were assessed by electrocardiography, echocardiography and treadmill tests, in addition to the evaluation of body weight, fasting glicemia, insulin and glucose tolerance at different time points. Histological analyses were performed in the heart tissue.

**Results:**

HFD consumption induced metabolic alterations characteristic of type 2 diabetes and obesity, as well as cardiac fibrosis and reduced exercise capacity. Upon returning to a standard diet, obese mice body weight returned to non-obese levels. G-CSF administration accelerated the reduction in of body weight in obese mice. Additionally, G-CSF treatment reduced insulin levels, diminished heart fibrosis, increased exercise capacity and reversed cardiac alterations, including bradycardia, elevated QRS amplitude, augmented P amplitude, increased septal wall thickness, left ventricular posterior thickening and cardiac output reduction.

**Conclusion:**

Our results indicate that G-CSF administration caused beneficial effects on obesity-associated cardiac impairment.

## Background

Diabetes is one of the most prevalent disorders and recent estimations suggest that there is a worldwide population of 347 million diabetic individuals [1]. According to WHO, diabetes will be the 7th leading cause of death in 2030 [2]. As the investigation of diabetes intensifies, it has become more evident that there exists a strong correlation between diabetes, obesity, high fat diet (HFD), sedentarism and cardiac abnormalities. With regards to diabetes-associated mortality and morbidity, the major culprit is alterations in cardiac structure and function. In this context, coronary arterial disease presents a higher incidence among heart alterations due to the diabetic condition that leads to heart failure. Diabetic cardiomyopathy is another cardiac disturbance which can be found independent of any trace of hypertension or ischemia [3], and represents an increased risk of heart failure in type 2 diabetes (DM2) patients. This disease was first described in four diabetic patients that died from heart failure, independent of an ischemic event or hypertension [4].

Excess fat consumption increases the risk of obesity and DM2, which can be followed by heart disease. The greater supply of fatty acid available increases its metabolism and oxidation, simultaneously reducing glucose uptake and oxidation, which represents more oxygen consumption by the heart without improving cardiac efficiency due to mitochondrial dysfunction [5–7]. DM2 and obesity are linked by different factors, such as production of pro-inflammatory cytokines and insulin resistance (IR) [8]. The inflammatory component of DM2 had also been demonstrated in experimental diabetes [9]. Several studies have suggested that the deterioration of cardiac function may be influenced by alterations in cytokine production, such as TGF-β, TNFα, IL-1β, IL-6 and IL-18 [10, 11], which participate in the formation of tissue fibrosis and inflammation. The hyperglycemia also worsens the cardiac function impairment due to an inflammatory process in the vascular endothelium, leading to micro- and macro-vascular complications [12–14].

Beneficial lifestyle changes, including a healthy diet and regular physical activity, contribute to the prevention of cardiovascular complications observed in obesity and DM2, however these positive life choices have yet to demonstrate functional cardiac recovery in the chronic diabetic state [15]. Recently, investigations have centered on the therapeutic use of granulocyte colony-stimulating factor (G-CSF), a cytokine known to promote the mobilization of bone marrow-derived hematopoietic stem cells into the circulation [16, 17]. G-CSF has shown beneficial effects on myocardial regeneration, such as the acceleration of wound healing, prevention of myocardial apoptosis and reduced myocardial fibrosis [18–21]. In addition, our laboratory has demonstrated in a mouse model of Chagas disease cardiomyopathy that treatment with G-CSF results in reduced fibrosis and inflammation in the heart, while improving electrocardiography (EKG) alterations and exercise capacity [22].

Based on our previous work, we hypothesized that G-CSF treatment would have beneficial effects in impaired cardiac function associated with obesity and DM2. In this study we investigated the therapeutic effect of G-CSF in a model of obese-diabetic C57Bl/6 mice generated by feeding with a HFD, which combines genetic susceptibility with environmental factors.

## Methods

### Experimental animals

Twenty weeks–old male C57Bl/6 mice were raised and maintained in the animal facilities at the Cell Therapy and Biotechnology Center of Hospital São Rafael (Salvador, Bahia, Brazil). Mice were housed at room temperature (20 ± 2 °C), under controlled humidity (50 %), with unrestricted access to food and water *ad libitum* and exposed a constant light–dark cycle of 12 h and 12 h. All procedures were approved by the Ethical Committee for Animal Research of CPqGM/FIOCRUZ.

### Induction of obesity and administration of G-CSF

C57Bl/6 mice were fed a standard mouse chow for up to five months of age. Mice were then divided into two groups: standard diet (Nuvital®, Paraná, Brazil) (*n* = 8) and HFD (Research Diets Inc; New Brunswick, NJ, USA) (*n* = 16) for 36 weeks to induce obesity. Thirty-six week exposure to HFD was selected based on a pilot study (our unpublished data) where we initially standardized the model of diabetic cardiomyopathy in C57BL/6 mice. At 36 weeks of HFD induction, significant cardiac abnormalities were detected by echocardiogram (ECHO) and EKG. Table 1 details the composition of the diets used in this study.

**Table 1:** Nutritional composition of high fat (HFD) and standard diets

HFD (D12330)			Standard diet		
	gm%	kcal%		gm%	kcal%
Protein	23.0	16.4	Protein	16.8	16.4
Carbohydrate	35.5	25.5	Carbohydrate	74.3	73.1
Fat	35.8	58.0	Fat	4.8	10.5
Total		100	Total		100
Kcal/gm	5.56		Kcal/gm	4.07	
Ingredients	gm	kcal	Ingredients	gm	kcal
Casein, 30 Mesh	228	912	Casein, 30 Mesh	228	912
DL-Methionine	2	0	DL-Methionine	2	0
Maltodextrin 10	170	680	Maltodextrin 10	170	680
Corn Starch	175	700	Corn Starch	835	3340
Sucrose	0	0	Sucrose	0	0
Soybean Oil	25	225	Soybean Oil	25	225
Coconut Oil, Hydrogenated	333.5	3001.5	Coconut Oil, Hydrogenated	40	360
Mineral Mix S10001	40	0	Mineral Mix S10001	40	0
Sodium Bicarbonate	10.5	0	Sodium Bicarbonate	10.5	0
Potassium Citrate, 1 H2O	4	0	Potassium Citrate, 1 H2O	4	0
Vitamin Mix V10001	10	40	Vitamin Mix V10001	10	40
Choline Bitartrate	2	0	Choline Bitartrate	2	0
FD&C Blue Dye #1	0.05	0	FD&C Yellow Dye #5	0.1	0
FD&C Red Dye #4	0.05	0			
Total	1000.1	5558.5		1366.6	5557

**Fig. 1:**
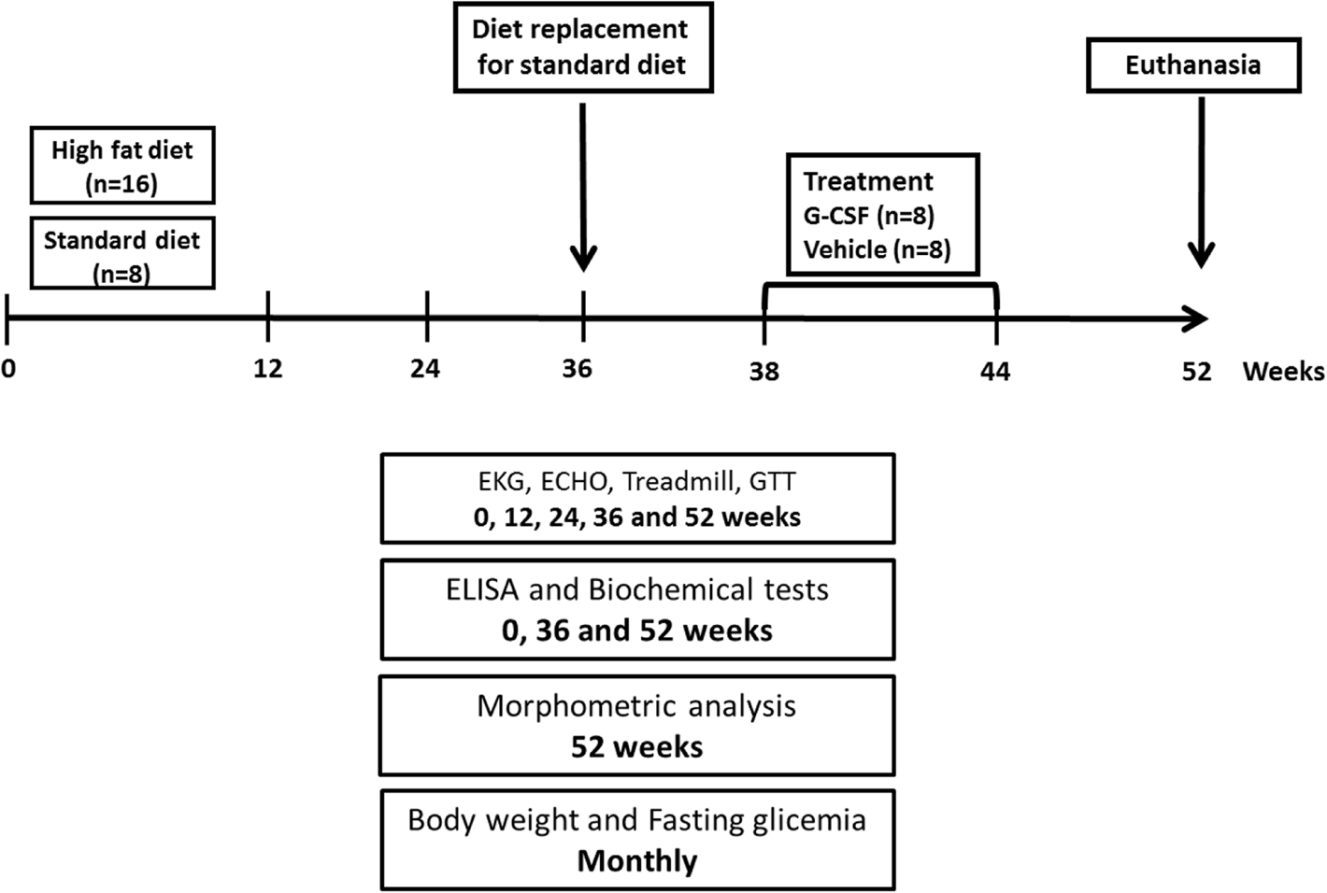
Experimental design. During the first 36 weeks, groups of C57Bl/6 mice were fed with HFD or standard diet. Subsequently, the HFD-fed mice were divided into two groups, receiving either G-CSF or vehicle (saline), while simultaneously returned to a standard diet. Functional testing (EKG, ECHO, treadmill) and glucose tolerance test (GTT) were done every 3 months. ELISA and biochemical tests were performed prior to and at the conclusion of the HFD administration protocol (36 weeks), as well as 8 weeks after the end of G-CSF/saline administration

Following 36 weeks of HFD consumption, obese mice were randomly divided into two sub-groups. The first group was treated with human recombinant G-CSF (Filgrastim - Bio Sidus S.A., Buenos Aires, Argentina) and vehicle (saline). The protocol of G-CSF treatment consisted of three courses of a daily injection (200 μg/kg/day in saline, intraperitoneally) for 5 days, including a 7-day interval between each course. The second group was the treatment control group that received equal volume of saline solution (100 μl i.p.) following the same injection protocol. When G-CSF and saline administration begun, obese mice were removed from HFD and replaced with a standard diet (Fig. 1).

### Assessment of body weight, glucose, insulin, total cholesterol and adiponectin

The induction of obesity was monitored by monthly measurements of body weight and fasting glucose levels, in addition to a quarterly evaluation of glucose tolerance, with samples collected from tail vein blood. The fasting glucose test was performed 7 h following the last food intake, while the glucose tolerance test was performed after a 4 h fasting. Tests were performed using an Accu-Chek Active glucometer system (Roche Diagnostics; Mannhein, Germany). Measurements of insulin, total cholesterol and adiponectin were performed prior to and at the conclusion of HFD administration protocol (36 weeks), as well as 8 weeks after to the conclusion of G-CSF administration. Insulin and total cholesterol evaluations were performed using a SBA 200 Celm (CELM Co.; São Paulo, Brazil). Adiponectin was measured in the plasma of mice by sandwich ELISA using DuoSet ELISA Development System kit (R&D Systems; Minneapolis, MN, USA). For these measurements, blood was collected from the orbital plexus under isoflurane anesthesia.

### Cardiac functional analysis

Assessment of cardiac function was performed every quarter. The cardiac function evaluation included: ECHO, EKG and treadmill test. For ECHO exam and EKG recordings, animals were anesthetized with inhaled isoflurane (0.5 %). Transthoracic echocardiography was performed on supine positioned mice maintained on a thermo-regulated plate (37 °C) to acquire images in different acoustic windows by using the Vevo 770 Ecosystem (Visual Sonics, Toronto, Canada) equipped with a 30 MHz transducer (Model 707B RMV, Visual Sonics).

ECHO analysis was performed using M-mode and B-mode image acquisition tools allowing for the visualization and measurement of left ventricular wall motion, anatomical structures, hemodynamics parameters, thereby enabling the detection of morphological and functional alterations, as described previously [23, 24]. Ventricular wall thickness and the inner diameter of the left ventricle during systole and diastole were determined from M-mode and B-mode images by measuring blood flow, using pulse Doppler hemodynamics. The function parameters evaluated were fractional shortening, ejection fraction, systolic volume, end-diastolic volume and cardiac output.

EKG acquisition was performed using a bipolar I lead, obtained from the Bio Amp PowerLab System (PowerLab 2/20; ADInstruments, Castle Hill, Australia), allowing for the recording of biological signals in animals (under isoflurane inhaled anesthesia) with complete electrical isolation. All acquired data was analyzed on Windows Chart 5 (PowerLab). Recordings were bandpass-filtered (1 to 100 Hz) to minimize environmental signal disturbances at a sampling rate of 1 kHz. The EKG analysis included heart rate, PR interval, P wave duration, QT interval, corrected QT Interval (QTc), and arrhythmias. Wave durations (ms) and heart rate were calculated automatically by the software. The QTc was calculated as the ratio of QT interval by square roots of RR interval (Bazett’s formula) [25].

A single-animal motor-driven treadmill chamber (LE 8700; Panlab, Barcelona, Spain) was used to exercise the mice. Treadmill speed and shock intensity (mA) were controlled by a potentiometer (LE 8700 treadmill control; Panlab). Room air was pumped into the enclosed compartment at a controlled flow rate (700 ml/min) by a chamber air supplier (Oxylet LE 400; Panlab). Mean room temperature was maintained at 21 ± 1 °C. After an adaptation period of 30 min in the treadmill chamber, mice were exercised at 5 different velocities (7.2, 14.4, 21.6, 28.8 and 36.0 m/min), with increasing velocity after 10 min of exercise at each given speed. Velocity was increased until the animal could no longer sustain a given speed and remained more than 10s on an electrified stainless-steel grid, which provided an electrical stimulus to maintain the mice in motion.

### Histopathological analysis

Hearts from control mice (fed with standard diet throughout the study), G-CSF-treated HF mice and vehicle-treated HF mice were removed and fixed in buffered 10 % formalin. Sections of paraffin-embedded tissue were stained with standard hematoxylin-and-eosin (H&E) and Sirius red for evaluation of inflammation and fibrosis, respectively, by optical microscopy. Images were digitized using a color digital video camera (CoolSnap, Montreal, Canada) adapted to a BX41 microscope (Olympus, Tokyo, Japan). The images were analyzed using Image Pro 5.0 (Media Cybernetics, San Diego, CA, USA), to integrate the number of inflammatory cells that were counted by area. Ten fields per heart were counted from each mouse/group. The percentage of fibrosis was calculated according to previously published methods [26]. Heart sections stained with Sirius red were digitized using a color digital video camera adapted to a BX41 microscope. Blinded analysis was performed on ten fields captured per heart, identifying areas of fibrosis and avoiding blood vessels (200× magnification). The percentage of fibrosis was estimated in each field using Image-Pro Plus software, where the percentage of the red area was compared with the remaining areas of the field. Results were presented as integrated area.

### Statistical analysis

Data were expressed as mean ± standard error of mean (SEM) for the number of animals in each group. Student’s *t* test was used to compare quantitative variables from the two groups at one point. When two or more groups were compared, analysis of variance (ANOVA) was applied, with the Newman-Keuls post-test. Significant difference was reached when p values were less than 0.05. Statistical analysis was performed with Graph Pad Prism 5.0 software (San Diego, CA, USA).

## Results

### G-CSF accelerates body weight recovery and reduces circulating insulin levels after HFD withdraw

**Fig. 2:**
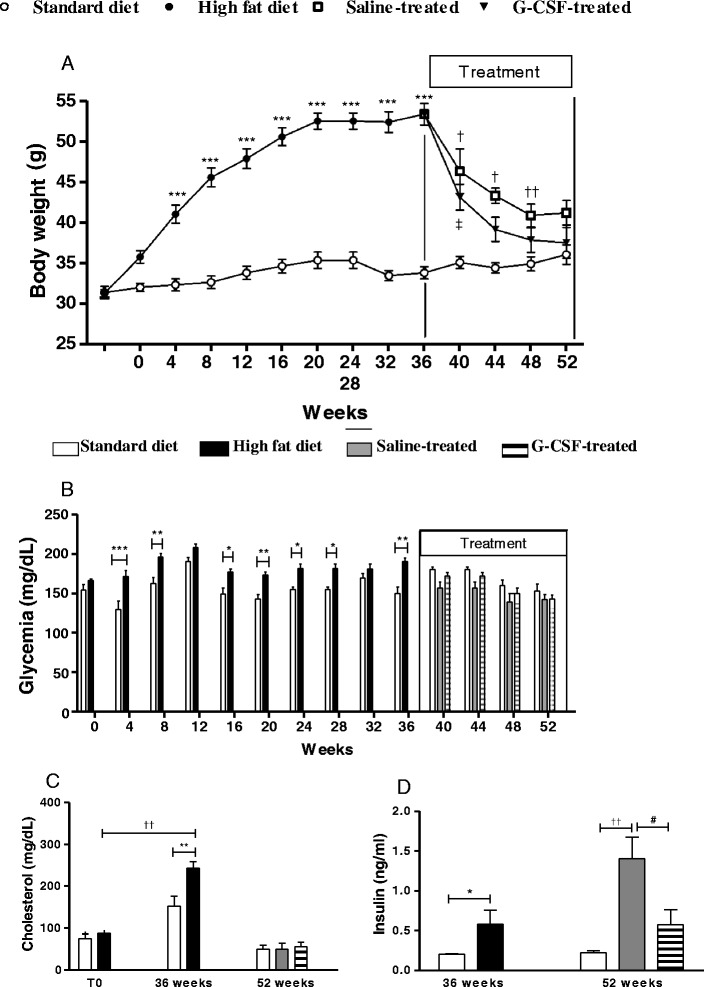
Body weight and biochemical analysis. **a** Body weight was measured from the beginning (T0) until the completion of HFD (36 weeks), in 4-week intervals. Following the completion of the HFD-induced DM2, mice returned to standard diet where some animals received G-CSF or saline treatment until 52 weeks. (Values are expressed as mean ± SEM. HFD mice vs. standard diet mice ***p < 0.001; G-CSF-treated mice vs. standard diet fed mice ‡*p* < 0.01; Saline-treated mice vs standard diet fed mice †*p* < 0.001; Saline-treated mice vs standard diet fed mice ††*p* < 0.001). (**b**) Glycemia was measured from the beginning (T0) until the completion of HFD (36 weeks), in 4-week intervals. Following the completion of the HFD-induced DM2, mice returned to standard diet where some animals received G-CSF or saline treatment until 52 weeks. (Values are expressed as mean ± SEM. HFD mice vs. standard diet mice, *p < 0.05, **p < 0.01, ***p < 0.001). **c** Cholesterol was measured before (T0), at the end of HFD consumption (36 weeks) and following G-CSF/saline administration (52 weeks) (Values are expressed as mean ± SEM. HFD mice vs. standard diet mice, **p < 0.01; G-CSF-treated mice T0 vs. T36, ††*p* < 0.01). **d** Plasma insulin concentrations were evaluated at 36 and 52 weeks. (Values are expressed as mean ± SEM. G-CSF-treated mice vs. standard diet mice *p < 0.05; Saline-treated mice vs standard diet mice, ††*p* < 0.001; HFD-Saline vs. HFD-G-CSF, #*p* < 0.05)

The introduction of HFD caused a significant increase in body weight compared to standard diet fed mice. At 36 weeks, HFD was withdrawn and all animals were fed a standard diet while received G-CSF or saline administrations (Fig. 1). Although the body weight from both HFD saline and HFD G-CSF groups consuming standard diet was normalized after 52 weeks, G-CSF treatment accelerated the weight loss (Fig. 2a). HFD resulted in an elevation in fasting glucose rates from the 8th to the 36th week following induction (Fig. 2b). Fasting glucose levels were significantly reduced following removal from HFD, however G-CSF did not influence this decrease (Fig. 2b). Similarly, HFD fed mice had significantly worse glucose tolerance test results when compared to standard diet fed mice (Table 2). Following the removal from HFD (week 36), no differences were observed in the glucose tolerance test at any time point, despite G-CSF administration (data not shown).

**Table 2:** Glucose tolerance test

	Standard	HFD	Standard	HFD	Standard	HFD	Standard	HFD	Standard	HFD
T0	133 ± 5.4	144 ± 5.2	314 ± 10.7	318 ± 13.9	261 ± 16.0	260 ± 10.0	209 ± 14.2	221 ± 8.7	162 ± 12.1	163 ± 7.6
12 w	163 ± 5.5	189 ± 4.6	294 ± 24.0	367 ± 14.9 **	233 ± 19.2	304 ± 13.0 ***	217 ± 6.6	303 ± 15.2 ***	196 ± 12.0	264 ± 15.9 **
24 w	140 ± 5.0	188 ± 7.4	300 ± 18.9	463 ± 17.1 ***	256 ± 18.6	389 ± 18.4 ***	187 ± 9.6	287 ± 18.7 ***	154 ± 7.9	208 ± 8.2 *
36 w	135 ± 7.2	150 ± 3.6	311 ± 19.1	413 ± 17.6 ***	231 ± 15.0	339 ± 21.7 ***	181 ± 8.4	265 ± 21.7 **	142 ± 11.7	204 ± 11.4 *
	0 min		15 min		30 min		60 min		120 min	

*p < 0.05, **p < 0.01, ***p < 0.001.

Total cholesterol was significantly elevated in HFD fed mice (at 36 weeks) compared to standard diet fed mice (Fig. 2c). Removal from HFD alone normalized cholesterol to levels to those observed in standard diet fed mice. In contrast, insulin levels, which were significantly elevated in HFD mice (36 weeks) did not normalize following removal from HFD (52 weeks). G-CSF administration caused a statistically significant reduction in insulin levels in HFD mice, reaching levels observed in control mice (Fig. 2d). Adiponectin was also measured at 36 and 52 weeks, however no statistical differences were detected between any of the groups evaluated (data not shown).

### G-CSF administration reverses cardiac function alterations in diabetic mice

**Fig. 3:**
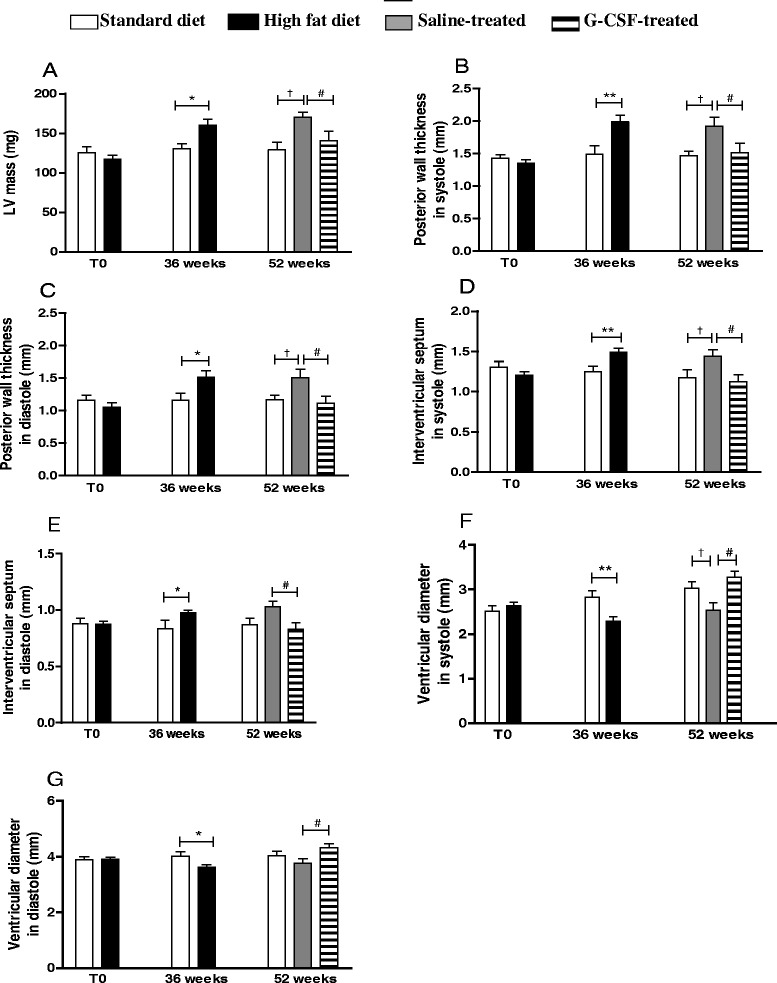
Echocardiography functional assessment. Echocardiographic analyses were performed before (T0), at the end of HFD consumption (36 weeks), and at 52 weeks. LV mass (**a**), posterior wall thickness during systole (**b**) and diastole (**c**), interventricular septum thickness during systole (**d**) and diastole (**e**), and LV internal diameter during systole (**f**) and diastole (**g**) were evaluated. Values are expressed as mean ± SEM. HFD mice vs. standard diet mice *p < 0.05, **p < 0.01. G-CSF-treated mice vs. saline-treated mice ^#^ *p* < 0.05. HFD-Saline mice vs. standard diet mice, †*p* < 0.05

The cardiac structural evaluation was performed by echocardiogram before and 36 weeks after HFD administration, as well as following G-CSF/saline administration at week 52. The parameters evaluated during systole and diastole were indicative of cardiac hypertrophy development in the HFD group, including increased left ventricle mass (Fig. 3a), posterior wall thickness (Fig. 3b and c) and septal thickness (Fig. 3d and e). Removal of HFD (at week 52) alone did not reverse these alterations, however, together with G-CSF administration, resulted in values similar to those observed in mice submitted to standard diet (Fig. 3a–e). Both systolic and diastolic left ventricle diameters from obese mice were significantly reduced, when compared to mice fed with standard diet (Fig. 3f and g). Treatment with G-CSF also reversed this alteration, causing a statistically significant increase in ventricular diameter during both systole and diastole.

**Fig. 4:**
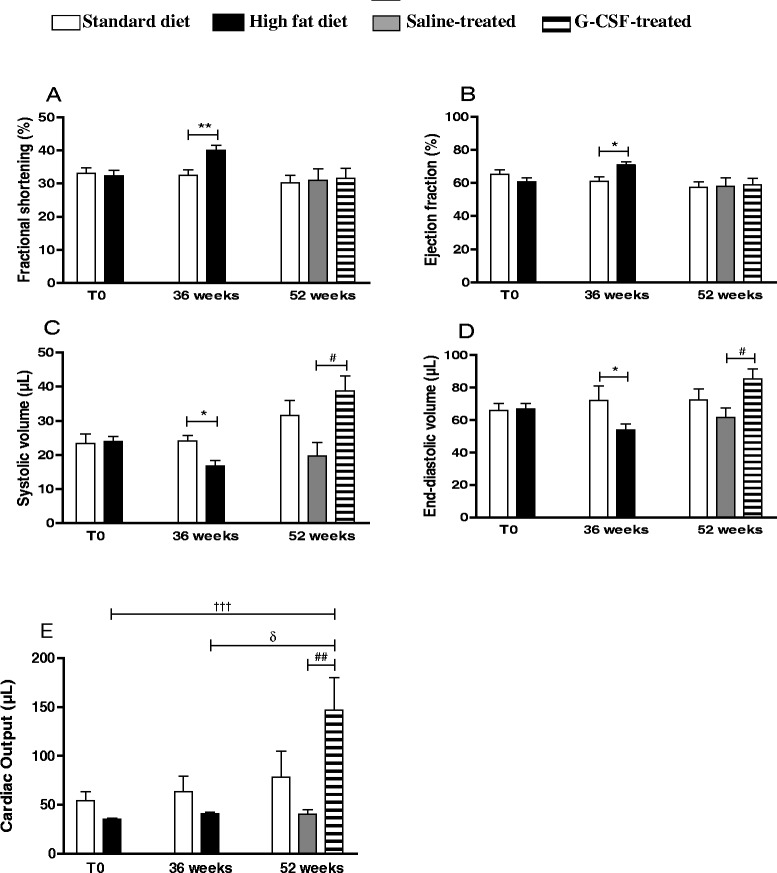
Echocardiography hemodynamic assessment. Echocardiographic analyses performed before and at the conclusion of DM2 induction (T0 and 36 weeks), and at 52 weeks. Fractional shortening (**a**), ejection fraction (**b**), systolic volume (**c**), end-diastolic volume (**d**) and cardiac output (**e**). Values are expressed as mean ± SEM. HFD mice vs. normal mice *p < 0.05, **p < 0.01. G-CSF-treated mice vs. saline-treated mice ^#^ *p* < 0.05. HFD mice (T0) vs. G-CSF-treated mice ^†††^ *p* < 0.01. HFD mice (36 weeks) vs. G-CSF-treated mice ^δ^ *p* < 0.05. For cardiac output (**e**), G-CSF-treated mice vs. saline-treated mice ^##^ *p* < 0.01, HFD mice (T0) vs. G-CSF-treated mice ^†††^ *p* < 0.01, HFD mice (36 weeks) vs. G-CSF-treated mice ^δ^ *p* < 0.05

Hemodynamic analyses revealed significant increases in fractional shortening (Fig. 4a) and ejection fraction (Fig. 4b) in obese mice after 36 weeks, however G-CSF administration did not have any additive effects on these two parameters after the HFD removal. Conversely, the mobilized volumes, such as systolic volume (Fig. 4c), end diastolic volume (Fig. 4d) and cardiac output (Fig. 4e) were significantly reduced in obese mice at the conclusion of HFD induction. Administration of G-CSF resulted in a statistically significant increase in both systolic and end-diastolic volumes, measured at week 52 (Fig. 4c and d). No differences were detected in isovolumetric relaxation time, or in isovolumetric contraction time, at 36 or 52 weeks. Removal of HFD alone normalized fractional shortening (Fig. 4a) and ejection fraction (Fig. 4b). G-CSF administration increased blood volume mobilized by the heart, as shown by the elevation of the end diastolic volume (Fig. 4d) and cardiac output (Fig. 4e).

**Fig. 5:**
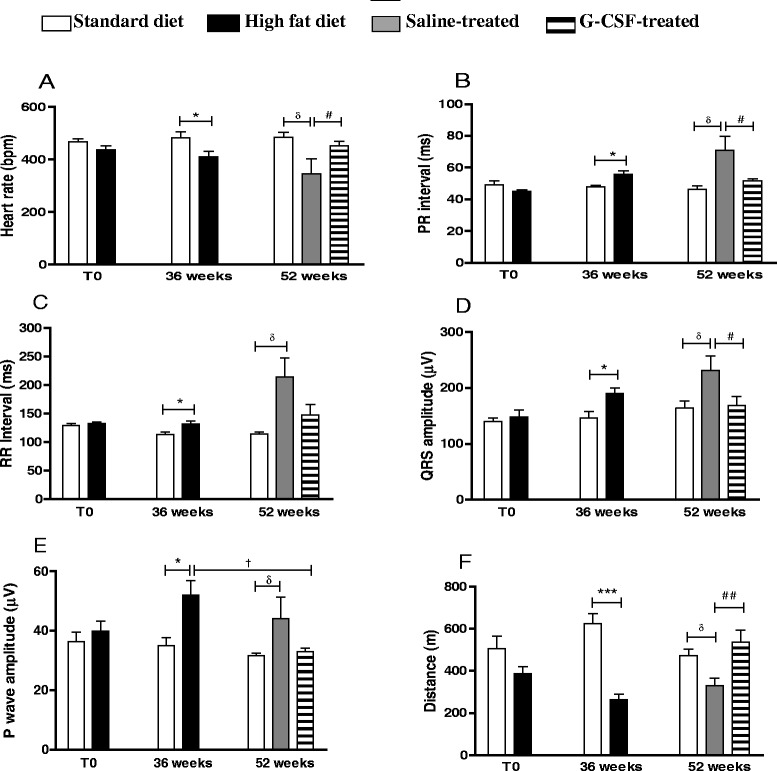
Electrocardiographic assessment and physical fitness evaluation. Electrocardiographic recording from mice before (T0), at the end of HFD consumption (36 weeks), and at 52 weeks. Altered parameters included: heart rate (**a**), PR interval (**b**), RR interval (**c**), QRS amplitude (**d**), P wave amplitude (**e**). Values are expressed as mean ± SEM. For **a**-**d**: HF mice vs. normal mice *p < 0.05, ***p < 0.001; HF treated mice vs. non-treated mice ^#^ *p* < 0.05, ^##^ *p* < 0.01; HFD mice (36 weeks) vs. G-CSF-treated mice ^†^ *p* < 0.05. (**e**) For P wave amplitude, HFD mice vs. standard diet fed mice *p < 0.05; Saline-treated mice vs. standard diet fed mice ^δ^ *p* < 0.05; HFD mice 36 weeks vs G-CSF-treated mice, ^†^ *p* < 0.05. (**f**) Animal performance during treadmill test was evaluated before (T0), at the end of HFD consumption (36 weeks), and at 52 weeks. Values are expressed as mean ± SEM. HFD mice vs. standard diet fed mice, ***p < 0.001; G-CSF-treated mice vs. saline-treated mice ^##^ *p* < 0.01; Saline-treated mice vs. standard diet fed mice, ^†^ *p* < 0.05

Thirty six weeks after HFD induction, obese mice heart rates was significantly reduced compared to control mice (Fig. 5a), while the PR and RR intervals, QRS amplitude and P wave were increased (Fig. 5b–e). Moreover, a significant reduction in running distance was observed in obese mice when compared to control, following the diet change at 36 weeks (Fig. 5f). EKG analysis showed a reversion of electrical alterations only after G-CSF administration, which normalized heart rate (Fig. 5a), PR interval (Fig. 5b), QRS (Fig. 5d) and P wave (Fig. 5e) amplitudes. Importantly, the physical exercise capacity was recovered in G-CSF-treated obese mice, as shown by the run distance (Fig. 5f).

In the present study, results evidenced LV hypertrophy in absence of diastolic or systolic alterations. However, no alterations were observed during the relaxation time or peak systolic velocity.

### Reversion of HFD-induced heart fibrosis in the heart after G-CSF administration

**Fig. 6:**
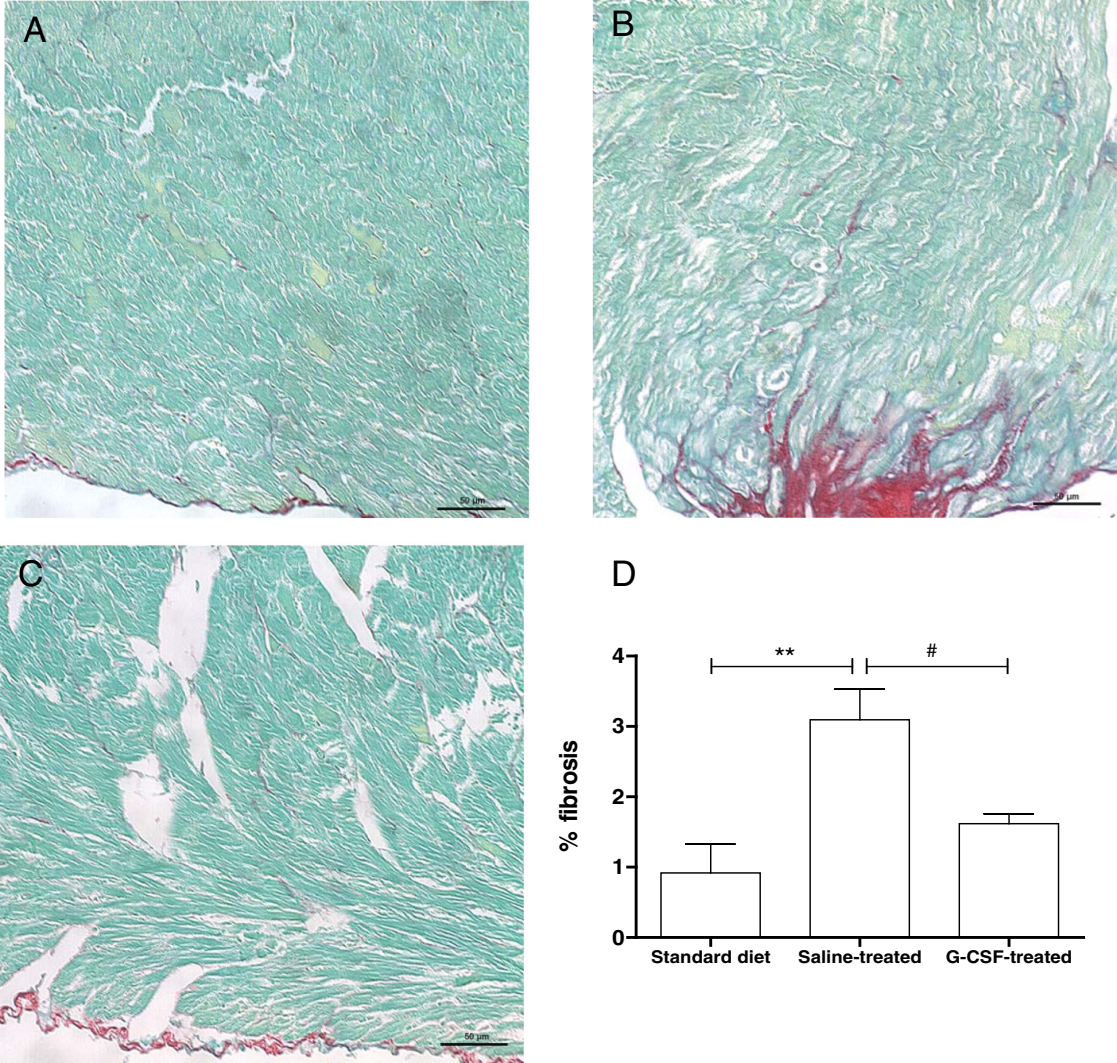
G-CSF administration reduces HFD-induced heart fibrosis. Heart sections of mice fed with standard diet (**a**), saline-treated obese mice (**b**) and G-CSF-treated obese mice (**c**). **d** Morphometric analysis of the percentage of fibrosis area in heart sections. Values are expressed as mean ± SEM. Standard diet mice vs. saline-treated mice **p < 0.01. Saline-treated mice vs. G-CSF-treated mice ^#^ *p* < 0.05. Calibration bars = 50 μm

Obese saline-treated mice had a significantly higher percentage of fibrosis in the heart at week 52, when compared to control mice (Fig. 6). G-CSF administration in obese mice caused a statistically significant reduction in fibrosis, when compared to saline-treated obese mice (Fig. 6), reaching levels similar to those of mice fed with standard diet. Inflammatory cells were not found in heart sections of any experimental group.

## Discussion

In the present study, we demonstrated that G-CSF administration contributed, at least partially, to the improvement of cardiac function in obese diabetic mice presenting concentric hypertrophy, characteristic of diabetic cardiomyopathy. This was reflected in the recovery of physical exercise capacity and the hypertrophy reversal measured by echocardiography. Additionally, we observed that G-CSF administration resulted in a reversion of fibrosis in the heart tissue, caused by HFD consumption.

The experimental strategy HFD removal was based on the American Diabetes Association recommendations that, at the onset of diabetes diagnosis, lifestyle changes (diet and exercise) with or without medication are needed to control blood glucose. Studies have demonstrated that the first step in treating DM2 is to limit the intake of saturated fatty acids, trans fatty acid and cholesterol, in order to reduce the cardiovascular risks [27–29]. Aligned with this recommendation, this study submitted mice to a HFD that resulted in an obese state, followed by returning to a standard diet accompanied by administration of G-CSF or saline.

DM2 and obesity are related and severely effect a large worldwide population [30–32]. Both conditions are associated with the development of hypertension, coronary disease, cardiomyopathy and micro- or macro-vascular injuries [33]. Diabetic cardiomyopathy was originally reported in diabetic patients, following heart failure-induced death while lacking evidence of hypertension, myocardial ischaemia or congenital or valvular heart disease [4]. Here, we used a model that presented pathophysiological characteristics of diabetic cardiomyopathy, using C57Bl/6 male mice fed with a HFD. This led to the induction of obesity-dependent diabetes, as well as myocardial disturbances, which resemble those found in humans.

Here we confirmed that the C57Bl/6 mouse strain is highly susceptible to develop DM2 following HFD exposure, also resulting in obesity, hyperglycemia and glucose intolerance, as previously reported [34–36]. After returning to a standard diet, and independent of G-CSF administration, glycemia levels were normalized to the levels of mice only fed with a standard diet. Our data is supported by previous studies demonstrating healthier dietary habits can result in positive outcomes for DM2 patients [37, 38]. Although removal from HFD resulted in a significant reduction in body weight, G-CSF administration caused an accelerated weight loss when compared to saline administration. Finally, G-CSF administration also resulted in an improved exercise capacity when compared to saline-treated obese mice.

Hyperinsulinemia is a component of the pathogenesis in obesity and diabetes. In the current study, saline administered obese-diabetic mice presented a significant level of hyperinsulinemia compared to non-obese-diabetic mice, even after returning to a standard diet. G-CSF administration significantly reduced circulating concentrations of insulin in obese-diabetic mice. To the best of our knowledge, this is the first time that G-CSF has been implicated in regulating insulin secretion. This reduction in insulin concentration induced by G-CSF administration may contribute to the accelerated weight loss observed in the obese-diabetic mice [39].

Anti-obesity effects of G-CSF have been demonstrated, resulting in the reduction of inflammatory cytokines that led to a loss in body weight in a model of obese-diabetic rats [40]. The anti-inflammatory effect of G-CSF is well characterized and has been observed in different disease models, including chronic Chagas disease cardiomyopathy, which has been shown in previous studies from our group [26]. Based on the effect of G-CSF on insulin regulation, the reduction of insulin and, potentially, a decrease in pro-inflammatory cytokines induced by G-CSF [41], may lead to a reduction in body weight, however further studies are needed to clarify this possibility.

The direct actions of G-CSF in modulating negative cardiac outcomes have been intensely investigated, which include reducing cardiac fibrosis. In the present study, we observed that G-CSF administration significantly reduced fibrosis in the heart tissue. Studies have suggested different mechanisms by which G-CSF reduces fibrosis, including modulation of synthesis and degradation of components in the extracellular matrix [42] as well as G-CSF-mediated signaling regulation of collagenase [43]. Our group has previously demonstrated the anti-fibrotic effects of G-CSF in the heart in a model of chronic Chagas disease cardiomyopathy, which results in progressive collagen deposition and fibrosis [26]. Therefore our current results in the obese-diabetic mouse model reinforce the anti-fibrotic effects of G-CSF.

In this study, the structural and hemodynamic parameters evaluated by ECHO suggested the development of concentric hypertrophy. The fractional shortening and ejection fraction were elevated in HFD mice compared to mice fed a standard diet, which is supported by clinical evidence of obesity-induced LV hypertrophy [44]. Moreover, the LV diameter was reduced in HFD mice, which was in accordance with the lowest end diastolic volume and systolic volume observed in this group, suggesting a reduction in LV compliance. The elevated P wave and QRS amplitudes recorded in EKG reflects the loss of LV compliancy and hypertrophy. G-CSF administration resulted in the reversal of the hypertophic state of the obese mouse heart, normalizing LV mass, posterior wall thickness (during both systole and diastole) and intraventricular septum thickness (during both systole and diastole) to levels of non-obese standard diet fed mice. One of the factors that may contribute to the recovery of compliance following G-CSF administration is the reduction of the fibrosis. Previous studies have demonstrated that G-CSF administration has protective effects against cardiac remodeling and acts to reduce cardiac hypertrophy [45, 46]. Thus, we suggest that G-CSF acts as a mediator against cardiac hypertrophy, presenting a promising therapeutic option for individuals with diabetic cardiomyopathy.

HFD increases apoptotic susceptibility that can lead to elevated cardiac insult vulnerabilities [47]. Increased apoptosis leads to hypertrophy [48] and fibrosis deposition in the myocardium [49] leading to cardiac dysfunction. G-CSF has anti-apoptotic properties on cardiomyocytes [50], and demonstrates anti-fibrotic activity [26]. Therefore, G-CSF can act as a protective mediator, leading to cardiac preservation and, potentially, reversal of heart damage.

## Conclusion

In summary, our results reinforce that G-CSF has a cardioprotective role, as well as acts as a modulator in diabetes and obesity. Evaluation of G-CSF during HFD administration needs to be performed in order to assess its effects during HFD consumption. Further studies are required in order to understand the molecular mechanisms involved in the protective actions of G-CSF.

## Abbreviations

WHO: World Health Organization
TGF-β: Transforming growth factor beta
TNF-a: Tumor Necrosis Factor-alpha
IL: Interleukin
DM2: Type 2 Diabetes mellitus
G-CSF: Granulocyte-colony stimulating factor
HFD: High fat diet
ELISA: Enzyme Linked Immuno Sorbent Assay
QTc: Corrected QT Interval
SEM: Standard error of mean
DC: Diabetic Cardiomyopathy
LV: Left ventricular

## References

[1] Danaei G, Finucane MM, Lu Y, Singh GM, Cowan MJ, Paciorek CJ (2011). National, regional, and global trends in fasting plasma glucose and diabetes prevalence since 1980: systematic analysis of health examination surveys and epidemiological studies with 370 country-years and 2.7 million participants. Lancet.

[2] Global status report on noncommunicable diseases 2010. Geneva, World Health Organization. 2011. http://www.who.int/nmh/publications/ncd_report_full_en.pdf.

[3] de Simone G, Devereux RB, Chinali M, Lee ET, Galloway JM, Barac A (2010). Diabetes and incident heart failure in hypertensive and normotensive participants of the Strong Heart Study. J Hypertens.

[4] Rubler S, Dlugash J, Yuceoglu YZ, Kumral T, Branwood AW, Grishman A (1972). New type of cardiomyopathy associated with diabetic glomerulosclerosis. Am J Cardiol.

[5] Akki A, Seymour AM (2009). Wesern diet impairs metabolic remodeling and contractile efficiency in cardiac hypertrophy. Cardiovasc Res.

[6] Pagano C, Calcagno A, Granzotto M, Calabrese F, Thiene G, Federspil G (2008). Eart lipid accumulation in obese non-diabetic rats: effect of weight loss. Nutr Metab Cardiovasc Dis.

[7] Buchanan J, Mazumder PK, Hu P, Chakrabarti G, Roberts MW, Yun UJ (2005). Reduced cardiac efficiency and altered substrate metabolism precedes the onset of hyperglycemia and contractile dysfunction in two mouse models of insulin resistance and obesity. Endocrinology.

[8] Eckel RH, Kahn SE, Ferrannini E, Goldfine AB, Nathan DM, Schwartz MW (2011). Obesity and type 2 diabetes: what Can Be unified and what needs to Be individualized?. Diabetes Care.

[9] Westermann D, Rutschow S, Jager S, Linderer A, Anker S, Riad A (2007). Contributions of inflammation and cardiac matrix metalloproteinase activity to cardiac failure in diabetic cardiomyopathy. Diabetes.

[10] Ko HJ, Zhang Z, Jung DY, Jun JY, Ma Z, Jones KE (2009). Nutrient stress activates inflammation and reduces glucose metabolism by suppressing AMPK in heart. Diabetes.

[11] Haugen E, Chen J, Wikstrom J, Grönros J, Gan LM, Fu LX (2007). Parallel gene expression of IL-6 and BNP during cardiac hypertrophy complicated with diastolic dysfunction in spontaneously hypertensive rats. Int J Cardiol.

[12] Avogaro A, Albiero M, Menegazzo L, De Kreutzenberg S, Fadini GP (2011). Endothelial dysfunction in diabetes: the role of reparatory mechanisms. Diabetes Care.

[13] Avogaro A, De Kreutzenberg SV, Fadini GP (2008). Oxidative stress and vascular disease in diabetes: is the dichotomization of insulin signaling still valid?. Free Radic Biol Med.

[14] Avogaro A, De Kreutzenberg SV, Fadini GP (2008). Endothelial dysfunction: Causes and consequences in patients with diabetes mellitus. Diabetes Res Clin Pract.

[15] Nonino-Borges CB, Borges RM, Santos JE (2006). Tratamento clínico da obesidade. Medicina.

[16] Delgaudine M, Gothot A, Beguin Y (2010). Spontaneous and granulocyte–colony-stimulating factor-enhanced marrow response and progenitor cell mobilization in mice after myocardial infarction. Cytotherapy.

[17] Link DC (2000). Mechanisms of granulocyte colonystimulating factor-induced hematopoietic progenitor-cell mobilization. Semin Hematol.

[18] Theiss HD, Gross L, Vallaster M, David R, Brunner S, Brenner C (2013). Antidiabetic gliptins in combination with G-CSF enhances myocardial function and survival after acute myocardial infarction. Int J Cardiol.

[19] Fazel S, Cimini M, Chen L, Li S, Angoulvant D, Fedak P (2006). Cardioprotective c-kit + cells are from the bone marrow and regulate the myocardial balance of angiogenic cytokines. J Clin Invest.

[20] Ott HC, Taylor DA (2006). From cardiac repair to cardiac regeneration – ready to translate?. Expert Opin Biol Ther.

[21] Kocher AA, Schuster MD, Szabolcs MJ, Takuma S, Burkhoff D, Wang J (2001). Neovascularization of ischemic myocardium by human bone-marrow-derived angioblasts prevents cardiomyocyte apoptosis, reduces remodeling and improves cardiac function. Nat Med.

[22] Macambira SG, Vasconcelos JF, Costa CRS, Klein W, Lima RS, Guimarães P (2009). Granulocyte colony-stimulating factor treatment in chronic Chagas disease: preservation and improvement of cardiac structure and function. FASEB J.

[23] Ghanem A, Röll W, Hashemi T, Dewald O, Djoufack PC, Fink KB (2006). Echocardiographic assessment of left ventricular mass in neonatal and adult mice: accuracy of different echocardiographic methods. Echocardiography.

[24] Stypmann J (2007). Doppler ultrasound in mice. Echocardiography.

[25] Berul CI, Aronovitz MJ, Wang PJ, Mendelsohn ME (1996). In vivo cardiac electrophysiology studies in the mouse. Circulation.

[26] Vasconcelos JF, Souza BS, Lins TF, Garcia LM, Kaneto CM, Sampaio GP (2013). Administration of granulocyte colony-stimulating factor induces immunomodulation, recruitment of T regulatory cells, reduction of myocarditis and decrease of parasite load in a mouse model of chronic Chagas disease cardiomyopathy. FASEB J.

[27] Pi-Sunyer FX, Maggio CA, McCarron DA, Reusser ME, Stern JS, Haynes RB (1999). Multicenter randomized trial of a comprehensive prepared meal program in type 2 diabetes. Diabetes Care.

[28] Evert AB, Boucher JL, Cypress M, Dunbar SA, Franz MJ, Mayer-Davis EJ, et al. Nutrition therapy recommendations for the management of adults with diabetes. Diabetes Care. 2013;36:3821–42. 10.2337/dc13-2042 PMC3816916 24107659

[29] van Horn L, McCoin M, Kris-Etherton PM, Burke F, Carson JA, Champagne CM (2008). Potential mechanisms and needs for future research are summarized for each relevant nutrient, food, or food component. J Am Diet Assoc.

[30] Bugger H, Abel ED (2014). Molecular mechanisms of diabetic cardiomyopathy. Diabetologia.

[31] Rato Q (2010). Diabetes mellitus: um problema de saúde global. Rev Port Cardiol.

[32] Cheng D (2005). Prevalence, predisposition and prevention of type II diabetes. Nutr Metab.

[33] Aronne LJ, Isoldi KK (2007). Overweight and obesity: Key components of cardiometabolic risk. Clin Cornerstone.

[34] Prpic V, Watson PM, Frampton IC, Sabol MA, Jezek GE, Gettys TW (2003). Differential mechanisms and development of leptin resistance in A/J versus C57BL/6 J mice during diet-induced obesity. Endocrinology.

[35] Noonan WT, Banks RO (2000). Renal function and glucose transport in male and female mice with diet-induced type II diabetes mellitus. Proc Soc Exp Biol Med.

[36] Rebuffé-Scrive M, Sutwit R, Feinglos M, Kuhn C, Rodin J (1993). Regional fat distribution and metabolism in a new mouse model (C57BL/6 J) of non-insulin-dependent diabetes mellitus. Metabolism.

[37] American Diabetes Association (2011). Standards of medical care in diabetes. Diabetes Care.

[38] Inzucchi SE, Bergenstal RM, Buse JB, Diamant M, Ferrannini E, Nauck M (2012). Management of hyperglycemia in type 2 diabetes: a patient-centered approach: position statement of the American Diabetes Association (ADA) and the European Association for the Study of Diabetes (EASD). Diabetes Care.

[39] Mehran AE, Templeman NM, Brigidi GS, Lim GE, Chu KY, Hu X (2012). Hyperinsulinemia drives diet-induced obesity independently of brain insulin production. Cell Metab.

[40] Lee Y, Song YS, Fang CH, So BI, Park JY, Joo HW (2014). Anti-obesity effects of granulocyte-colony stimulating factor in Otsuka-Long-Evans-Tokushima fatty rats. PLoS One.

[41] Linden MA, Pincu Y, Martin SA, Woods JA, Baynard T (2014). Moderate exercise training provides modest protection against adipose tissue inflammatory gene expression in response to high-fat feeding. Phys Rep.

[42] Minatoguchi S, Takemura G, Chen XH, Wang N, Uno Y, Koda M (2004). Acceleration of the healing process and myocardial regeneration may be important as a mechanism of improvement of cardiac function and remodeling by postinfarction granulocyte colony-stimulating factor treatment. Circulation.

[43] Sugimoto C, Fujieda S, Sunaga H, Noda I, Tanaka N, Kimura Y (2001). Granulocyte colony-stimulating factor (G-CSF)-mediated signaling regulates type IV collagenase activity in head and neck cancer cells. Int J Cancer.

[44] Woodiwiss AJ, Libhaber CD, Majane OH, Libhaber E, Maseko M, Norton GR (2008). Obesity promotes left ventricular concentric rather than eccentric geometric remodeling and hypertrophy independent of blood pressure. Am J Hypertens.

[45] Szardien S, Nef HM, Voss S, Troidl C, Liebetrau C, Hoffmann J (2012). Regression of cardiac hypertrophy by granulocyte colony-stimulating factor-stimulated interleukin-1β synthesis. Eur Heart J.

[46] Huber BC, Beetz NL, Laskowski A, Ziegler T, Grabmaier U, Kupatt C (2015). Attenuation of cardiac hypertrophy by G-CSF is associated with enhanced migration of bone marrow-derived cells. J Cell Mol Med.

[47] Littlejohns B, Pasdois P, Duggan S, Bond AR, Heesom K, Jackson CL (2014). Hearts from mice Fed a Non-obesogenic high-Fat diet exhibit changes in their oxidative state, calcium and mitochondria in parallel with increased susceptibility to reperfusion injury. PLoS One.

[48] Van Empel VP, De Windt LJ (2004). Myocyte hypertrophy and apoptosis: a balancing act. Cardiovasc Res.

[49] Harvey PA, Leinwand LA (2011). The cell biology of disease: cellular mechanisms of cardiomyopathy. J Cell Biol.

[50] Li Y, Takemura G, Okada H, Miyata S, Esaki M, Maruyama R (2006). Treatment with granulocyte colony-stimulating factor ameliorates chronic heart failure. Lab Invest.

